# BKV activates the interferon-β response in primary and immortalized microvascular endothelial cells late in the infectious cycle, resulting in persistence

**DOI:** 10.1128/jvi.00934-26

**Published:** 2026-08-31

**Authors:** Lindsay C. Colgan, Ping An, Paul G. Cantalupo, Wenshan Zheng, Abigail R. Velasquez, Alexis M. Duray, Maria Teresa Sáenz Robles, David Weitz, James M. Pipas

**Affiliations:** 1 Department of Biological Sciences, University of Pittsburgh 171653, Pittsburgh, Pennsylvania, USA; 2 Department of Biomedical Informatics, University of Pittsburgh School of Medicine 198575, Pittsburgh, Pennsylvania, USA; 3 John A. Paulson School of Engineering and Applied Sciences, Harvard University 124077 , Cambridge, Massachusetts, USA; 4 Department of Chemistry and Chemical Biology, Harvard University 164189, Cambridge, Massachusetts, USA; 5 Department of Physics, Harvard University 242596, Cambridge, Massachusetts, USA; 6 Wyss Institute for Biologically Inspired Engineering, Harvard University 465574, Boston, Massachusetts, USA; Tufts University, Boston, Massachusetts, USA

**Keywords:** endothelial cells, innate immunity, polyomavirus BKV

## Abstract

Our previous studies have shown that inoculation of primary endothelial cells with BKV results in a long-term persistent infection, with inoculated cultures surviving up to 60 days while producing low levels of infectious viral progeny. Unlike the permissive renal proximal tubule cells, infected endothelial cells activate innate immunity pathways in response to BKV infection. We hypothesized that the innate immune response limits BKV infection, resulting in persistence. In this report, we show that activation of innate immune pathways is not triggered by incoming virus particles but rather occurs at a late stage of the infectious cycle, when viral DNA replication, high levels of late viral transcription, and progeny virion assembly are occurring. Single-cell transcriptomic data indicate that this response varies across the infected cell population, with some cells showing little or no innate immune response, while others appear to be highly activated. Bulk RNA-seq experiments show that the addition of interferon-β to endothelial cells results in the upregulation of a subset of known interferon-stimulated genes (ISGs), as well as many other genes not known to be associated with the interferon response. Finally, we show that the addition of interferon-β blocks BKV infection, so long as it is added prior to or soon after inoculation. These results lead to a model in which BKV infection of endothelial cells results in interferon production and ISG induction that restrict subsequent rounds of infection and lead to a persistent state.

**IMPORTANCE:**

BKV infects nearly every human on Earth, and the infection persists throughout life. The cell types where the virus persists remain a mystery, as do the state of the viral genome during persistence, as well as the mechanisms that limit productive infection and maintain persistence. While BKV is known to replicate robustly in renal proximal tubule cells during disease, it is not clear whether the virus persists in these cells. The proximal tubules are located in close proximity to endothelial cells, raising the possibility that BKV persistence occurs in the endothelia, and virus particles from infected endothelia potentially seed infections in the neighboring tubular epithelia during times of cellular stress or immunosuppression. The development of cell culture systems to study the interactions between innate immunity, virus production, and persistence is needed to advance the development of anti-viral strategies.

## INTRODUCTION

Human polyomavirus 1 (HuPyV1), also known as BK virus (BKV), is a member of the Polyomaviridae, a family of small viruses with circular double-stranded DNA genomes. The genomes of all polyomaviruses consist of three elements: a noncoding regulatory region (NCRR) that contains transcriptional promoters and the origin of viral DNA replication; an early transcriptional unit that encodes a large T antigen (LT) and a small t antigen (ST); and a late transcriptional unit that encodes the viral capsid proteins (1, 2). Nearly the entire human population is infected with BKV, and infection can persist throughout life (3). Usually, BKV is undetectable in healthy individuals; however, the virus is occasionally secreted in urine (4, 5). Immunosuppressed individuals sometimes secrete large amounts of BKV, and the virus is associated with diseases, including nephropathy and interstitial cystitis, especially in transplant patients undergoing immunosuppressive therapy (6–8). The tissues and cell types that harbor reservoirs of BKV and the mechanisms that govern the switch from low-level persistence to robust productive infection are unknown.

BKV is frequently observed in proximal and distal tubular epithelial cells in patients with nephropathy (9, 10). Cultured renal proximal tubule epithelial (RPTE) cells support robust productive infection with BKV, suggesting that these are the major permissive cell type in humans (11, 12). However, it has been shown that BKV can infect many human cell types, and the response to infection is cell type–specific (13–19). BKV establishes a persistent infection in cultured primary endothelial cells, and these cells have an activated innate immune response. Infected endothelial cells exhibit an interferon (IFN) response, including interferon-beta expression, STAT1 phosphorylation, and the production of many interferon-stimulated genes (ISGs) (13). We hypothesize that the activation of the innate immune response limits BKV infection of endothelial cells, resulting in persistence. In this manuscript, we show that immortalized human endothelial cells behave similarly to primary cells in terms of mounting an interferon response to BKV infection and the establishment of persistence, and we characterize endothelial cell infection at the single-cell level.

## RESULTS

### BKV infects primary and immortalized endothelial cells

**Fig 1:**
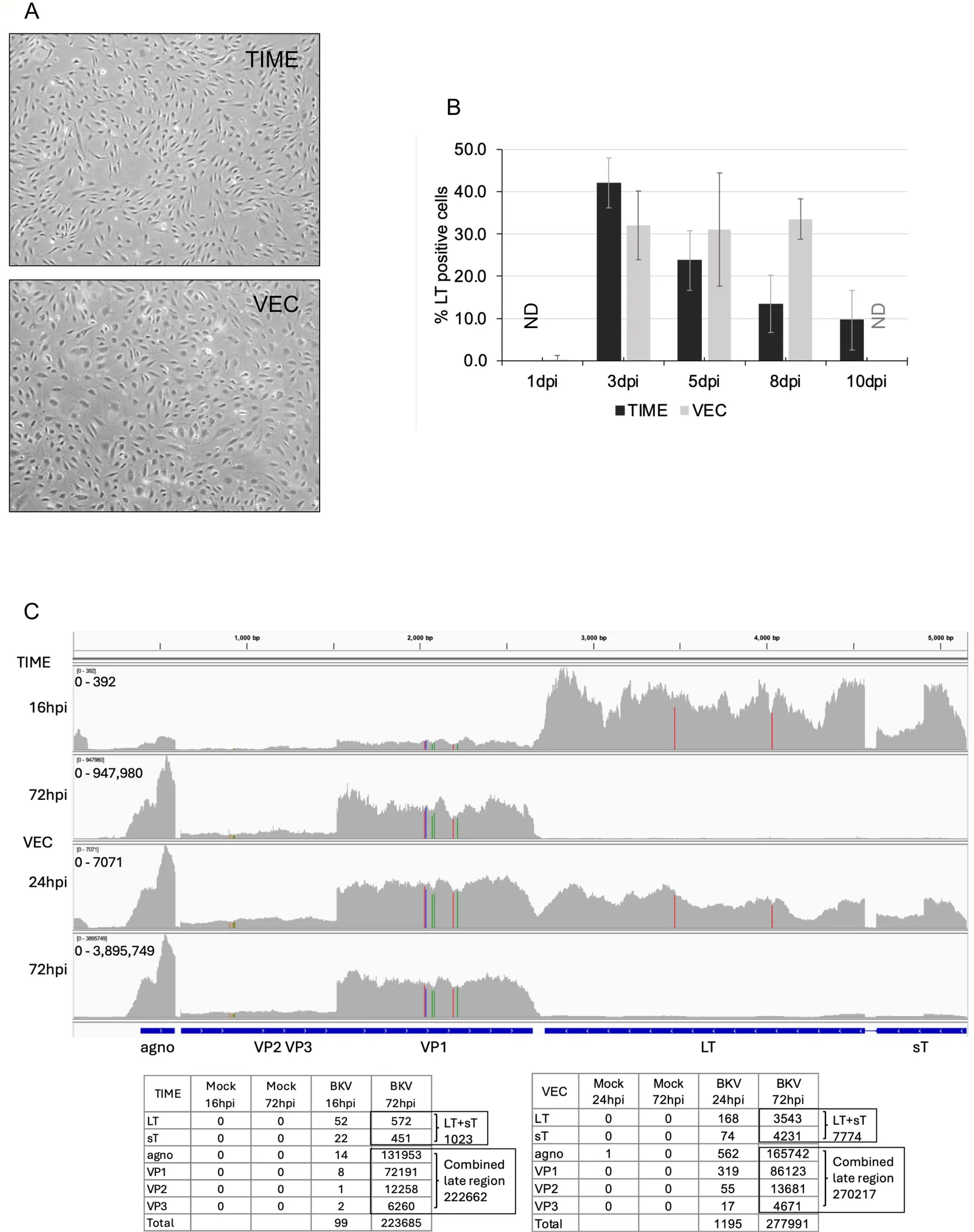
BKV infected both vascular endothelial cell (VEC) and TIME cultures. (**A**) TIME and VEC cultures display a similar morphology. (**B**) LT expression was determined by IF in BKV-infected TIME and VEC. The graph shows quantification of percent LT-positive cells in BKV-inoculated TIME and VEC at various time points in dpi. Each error bar represents the standard deviation of 3–4 measurements from a representative experiment. ND, not determined for TIME at 1 dpi and VEC at 10 dpi. (**C**) BKV early and late RNA expression based on bulk RNA-seq using infected TIME and VEC. RNA-seq coverage across the BKV genome (NC_001538.1) was plotted using Integrative Genomics Viewer (IGV). Gray peaks represent read depth at each position. Colored bars indicate positions that differ from the reference genome. BKV gene annotations are shown at the bottom. Top panels show coverage maps at different time points as indicated on the left. hpi, hours post-inoculation. Tables below the coverage maps summarize TPM (Transcripts per million) values of viral transcripts in mock- and BKV-infected TIME and VEC.

Primary microvascular endothelial cells from human lung tissue and human telomerase reverse transcriptase (hTERT)-immortalized microvascular endothelial cells (TIME) of dermal origin exhibit a similar morphology in culture (Fig. 1A). To assess their response to BKV infection, both cell types were infected at a multiplicity of infection (MOI) of 5, and LT protein expression was analyzed by immunofluorescence (IF). LT expression was detected in both cell types at 3 days post-inoculation (dpi) and remained detectable through the endpoint of the experiment (Fig. 1B). In both cases, a subset of inoculated cells (10%–40%) stained positive for LT, indicating that the majority of cells had little or no early viral gene expression.

Next, we assessed viral and cellular gene expression by performing bulk RNA-seq on mock and infected VEC and TIME cultures. Viral gene expression was assessed by comparing TPM values of BKV early (LT and ST) and late (Agno, VP1, VP2, and VP3) genes at two time points for each cell type (Fig. 1C). As expected, early region viral transcripts dominate at 16–24 h post-infection, while late viral transcripts are more abundant at 72 h post-infection. This pattern is indicative of a typical productive infection. Given that immunostaining for LT was observed in less than half the cell population, we conclude that full productive infection occurred in a subset of the inoculated cells.

### BKV induces an interferon pathway response in primary and immortalized endothelial cells

**Fig 2:**
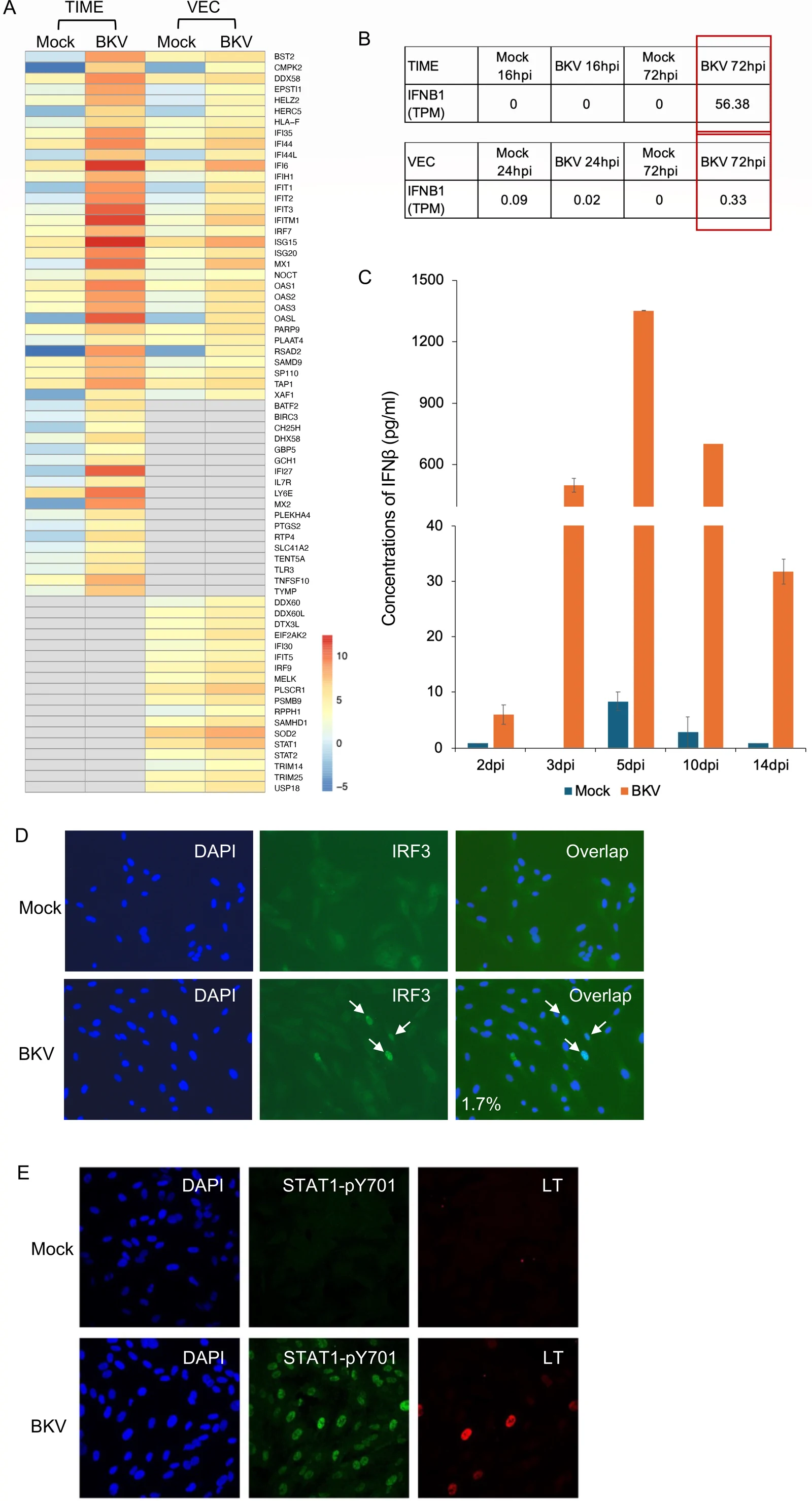
BKV infection led to activation of the IFN response. (**A**) BKV infection upregulated the expression of ISGs in TIME and VEC based on bulk RNA-seq. A heatmap was generated using the log_2_ TPM values of the top 50 ISGs in both cell types at 72 hpi. (**B**) TIME and VEC produced IFNβ mRNA in response to BKV infection. TPM values were determined from bulk RNA sequencing. (**C**) BKV-infected TIME secreted IFNβ into the culture supernatants. IFNβ concentrations were measured using culture supernatants of mock- and BKV-inoculated cells at different time points in pg/mL. (**D**) BKV activates nuclear translocation of IRF3 in TIME at 2 dpi. Quantification of percent IRF3-positive cells by overlapping IRF3 and DAPI immunofluorescence signals (bottom right image) was indicated. (**E**) BKV infection led to activation of STAT1 and nuclear translocation of phosphorylated STAT1 in TIME at 2 dpi. IF staining detected STAT1-pY701 (in green) and LT (in red) in the nuclei (DAPI, in blue) of TIME infected with BKV.

BKV infection in VEC triggers a type I IFN response characterized by the induction of multiple ISGs (13). A similar induction of ISGs was observed in BKV-infected TIME cells, as evidenced by bulk RNA-seq (Fig. 2A). In both primary and immortalized endothelial cells, BKV infection led to the induction of approximately 70 ISGs, with 32 ISGs induced in both. Generally, ISG induction levels were higher in BKV-infected TIME cells. IFNβ transcripts were detected at 72 hpi in both VEC and TIME, with a notably higher level in TIME (Fig. 2B). IFNβ secretion was confirmed by ELISA (Fig. 2C), which detected IFNβ in infected TIME culture supernatants from 2 to 14 dpi, with the peak level at 1,352 pg/mL at 5 dpi. To further verify the signaling events involved in the IFN response, immunofluorescence was used to demonstrate nuclear translocation of IRF3 (Fig. 2D), a key transcription activator of IFNβ, and phosphorylated STAT1, which is crucial for the activation of ISG transcription (Fig. 2E). Notably, IFN gene expression or protein secretion was not detected at early times post-infection. Rather, the induction occurred late in the infectious cycle after the onset of viral DNA replication and late gene expression.

### BKV infection persists in primary and immortalized endothelial cells

**Fig 3:**
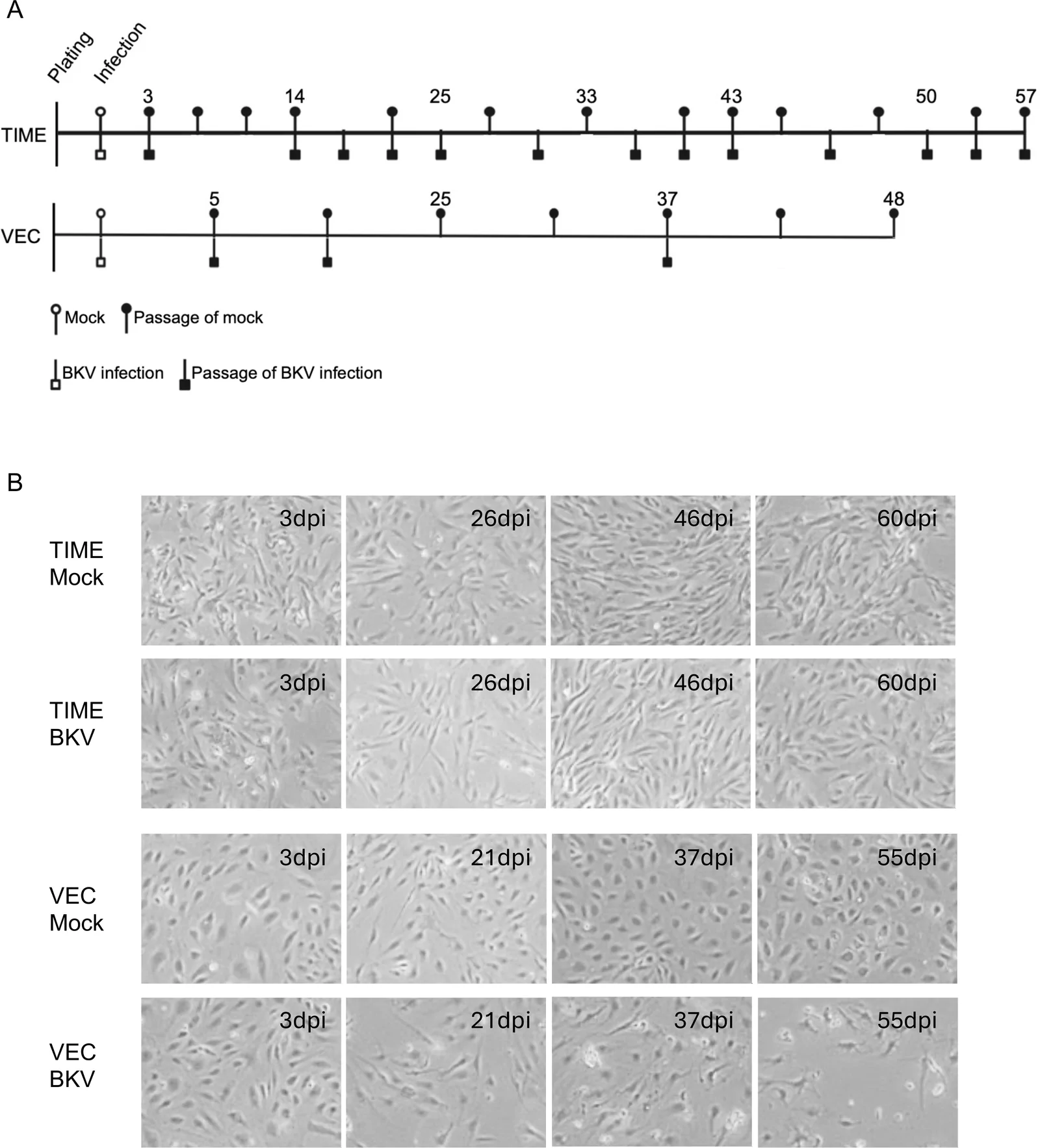
Persistent BKV infection was tracked in TIME and VEC for up to 60 days. (**A**) A timeline of BKV persistence experiments in TIME and VEC. Numbers indicate dpi. (**B**) Representative pictures showing typical cell morphology of mock- and BKV-infected TIME and VEC at different time points throughout the persistent infection experiments.

Our previous study demonstrated that BKV infection persisted for up to 66 days in primary human lung endothelial cells (13). To evaluate BKV persistence in immortalized endothelial cells, TIME cells were infected and maintained in culture alongside mock-infected controls. Both BKV-infected and mock-infected cells were passaged upon reaching confluency. For comparison, the same infection-passage experiment was conducted in parallel using VEC. Mock and infected VEC cultures showed signs of senescence at the later time points. In contrast, TIME cultures remained healthy and proliferative throughout the experiment. Consequently, BKV-infected TIME cells were passaged 13 times over a 60-day period post-infection, whereas BKV-infected primary VEC cultures were passaged only 3 times over 55 days (Fig. 3A).

**Fig 4:**
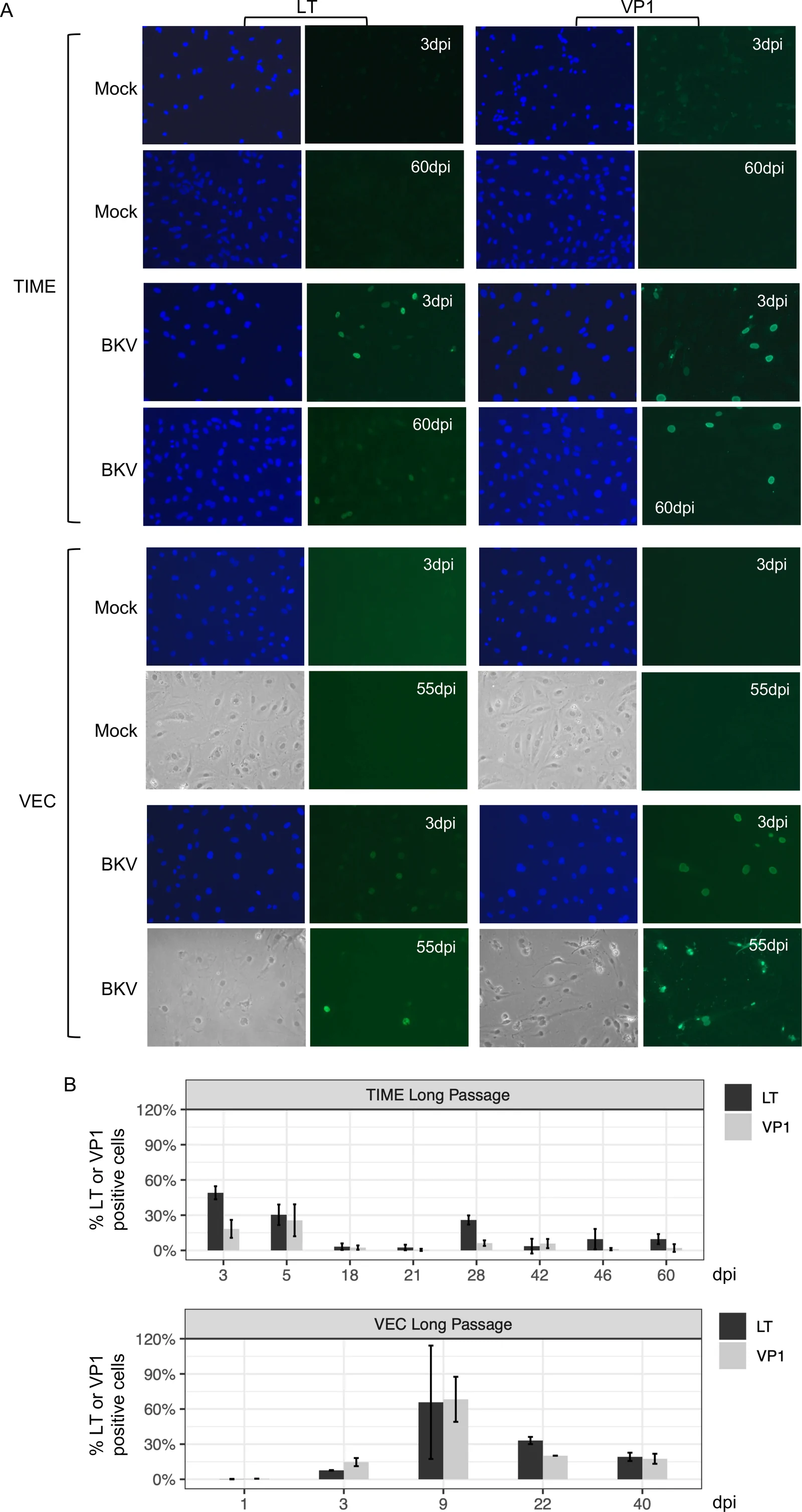
Persistent infection of TIME and VEC by BKV resulted in continuous viral protein expression. (**A**) Representative pictures showing LT and VP1 protein expression determined by IF at 3 dpi and the endpoints of the persistence experiments (60 dpi in TIME and 55 dpi in VEC). (**B**) Quantification of % LT- and VP1-positive cells in BKV-inoculated cells based on IF. Each error bar represents the standard deviation of 3–4 measurements from a representative experiment.

The expression of viral proteins, viral DNA replication, and the production of infectious progeny were monitored at various time points during the experiment. Morphologically, mock- and BKV-infected TIME cells appeared largely indistinguishable throughout the experiment (Fig. 3B). In contrast, mock- and BKV-infected VEC displayed notable differences between the early and late stages, with late-stage VEC, particularly BKV-infected VEC, exhibiting an enlarged and extended morphology characteristic of senescent cells. These cells also failed to reach confluency (Fig. 3B and 4A).

Results of immunostaining for LT and VP1 proteins throughout the long passage experiments are summarized in Fig. 4. LT and VP1 protein expression was detected at 3 dpi in the BKV-infected TIME and VEC and remained detectable until the termination of the experiments. In BKV-infected TIME cells, LT expression fluctuated between 18 and 60 dpi, with the percentage of LT-positive cells ranging from 2.5% to 26%. In contrast, the percentage of VP1-positive cells remained below 7%, suggesting the maintenance of a low-level productive infection (Fig. 4B). Quantification of LT and VP1 expression in BKV-infected VEC was performed at the 22 and 40 dpi time points, following the initial peak of viral protein expression at 9 dpi. At both time points, the percentage of VP1-positive cells exceeded 15% (Fig. 4B), which was higher than that observed in BKV-infected TIME (< 7%, Fig. 4B).

**Fig 5:**
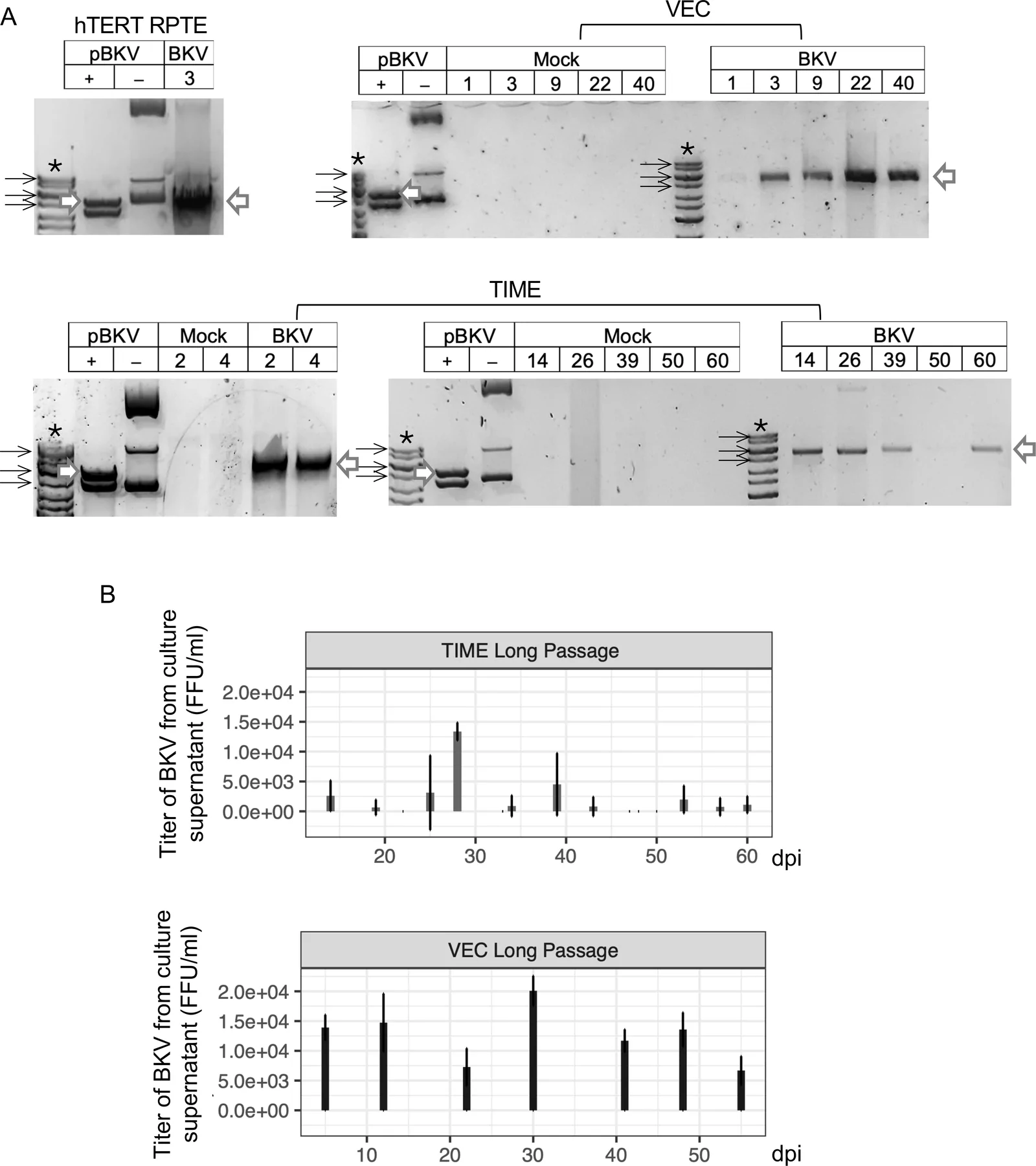
BKV persistence in TIME and VEC resulted in prolonged viral DNA replication and production of infectious progeny. (**A**) Viral DNA was detected in BKV-infected TIME (up to 60 dpi) and VEC (up to 40 dpi) at different time points of the persistence experiments. Due to the various time points assayed and multiple controls included, DNA samples from mock and BKV-infected cells as well as controls (pBKV plasmid and infected hTERT RPTE [hTERT immortalized human renal proximal tubular epithelial cells]) were analyzed across multiple runs of agarose gel electrophoresis. Each agarose gel image includes BamHI-digested BKV genomic DNA isolated at different time points along with the digested (+) and undigested (−) pBKV control plasmid. Cell types were indicated above the gel images (upper left, hTERT RPTE as cell type controls; upper right, VEC; bottom, TIME). The * indicates lanes of 1 kb DNA ladder. The bands corresponding to 10, 6, and 5 kb are indicated by sets of three arrows stacked from top to bottom. White block arrows with gray outline indicate linearized viral genomic DNA. Numbers indicate dpi. (**B**) BKV-infected TIME and VEC produced infectious progeny that were released into the culture supernatant. The titer of progeny BKV was determined by inoculating hTERT RPTE4 with BKV-infected TIME and VEC culture supernatants collected during the persistence infection. The x-axis indicates the time points for collection of supernatants. Each error bar represents the standard deviation of 3–4 measurements from a representative experiment.

The persistence of BKV viral DNA, observed as a single band migrating at approximately 5 kb on agarose gels, was evident in BKV-infected TIME and VEC. No viral DNA was detected in mock-infected controls throughout the experiment (Fig. 5A). A consistent amount of pBKV plasmid was loaded in each gel as a reference for comparing viral DNA levels across samples. In BKV-infected TIME, viral DNA was detectable up to 60 dpi, with the only exception at 50 dpi, indicating sustained viral replication and maintenance over an extended period. In VEC, viral DNA persisted up to 40 dpi, which was the last available time point for sample collection due to insufficient cell numbers. The intensity of the DNA bands suggested that BKV DNA levels peaked at 22 dpi in VEC, whereas in TIME, peak viral DNA levels were observed much earlier, between 2 and 4 dpi (Fig. 5A). Both TIME and VEC cells released infectious BKV progeny into the culture supernatant (Fig. 5B). In BKV-infected TIME, the levels of infectious progeny remained generally low (<5 × 10^3^ FFU/mL) except for a notable increase of 1.3 × 10^4^ FFU/mL at 28 dpi across the 10 time points tested. In contrast, BKV-infected primary VEC released higher levels of progeny, with titers greater than 1 × 10^4^ FFU/mL at 5 out of 7 time points. We conclude that BKV establishes a long-term persistent infection in TIME cultures, similar to that seen with primary VEC.

### Interferon-β treatment blocks BKV infection of endothelial cells

**Fig 6:**
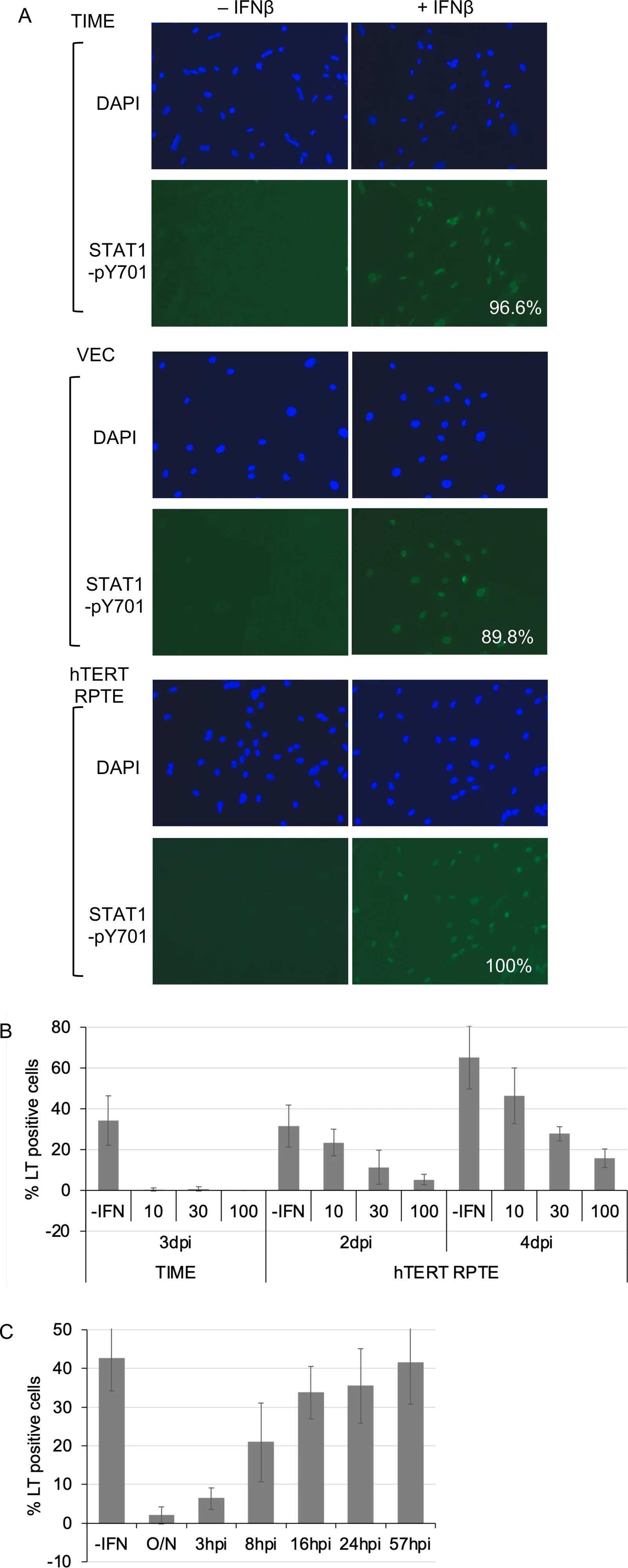
IFNβ blocked BKV infection. (**A**) Nuclear translocation of STAT1 in TIME, VEC, and hTERT RPTE treated with IFNβ. IF staining using STAT1-pY701 antibody shows STAT1 nuclear localization in cells treated with 100 U/mL IFNβ for 3 hours before fixing. (**B**) Pre-treatment with 10 IU/mL, 30 IU/mL, and 100 IU/mL IFNβ inhibits LT expression in BKV-infected TIME and hTERT RPTE. Quantification of percent LT-positive cells was done following IF. Each error bar represents the standard deviation of five measurements from a representative experiment. (**C**) IFNβ inhibits the early stage of BKV infection in TIME. 100 IU/mL of IFNβ was added to the cell culture media at different time points indicated by the x-axis (−IFN, no IFNβ was added as a negative control; O/N, overnight pre-treatment with IFNβ before inoculation). Quantification of % LT-positive cells in BKV-infected TIME at 3 dpi was determined by IF. Each error bar represents the standard deviation of five measurements from a representative experiment. O/N, overnight pre-inoculation treatment of IFNβ.

To investigate potential contributions of IFNβ to limiting BKV infection, TIME and hTERT RPTE (hTERT immortalized human renal proximal tubular epithelial cells) were treated with IFNβ at various time points before and/or after the inoculation. Immunostaining with the STAT1-pY701 antibody revealed robust nuclear localization in TIME and VEC treated with 100 IU/mL IFNβ for 3 hours, indicating that the IFNβ signaling pathway was effectively activated in both cell types (Fig. 6A). While pre-treatment with varying concentrations of IFNβ (10, 30, and 100 IU/mL) led to a dose-dependent reduction in LT expression in BKV-infected hTERT RPTE cells, pre-treatment with the lowest dose of IFNβ, 10 IU/mL, resulted in a near complete block of BKV infection shown by the lack of LT expression in the inoculated TIME (Fig. 6B). We conclude that treatment of primary and immortalized endothelial cells, as well as hTERT-RPTE, with IFNβ prior to inoculation inhibits BKV infection.

In a separate experiment, IFNβ was added to the culture media at different time points relative to BKV inoculation. Results showed that the timing of treatment significantly influenced its efficacy in suppressing LT expression (Fig. 6C). Pre-treatment with IFNβ overnight (O/N) prior to inoculation and treatment at 3 hours post-inoculation resulted in the most substantial reduction in LT-positive cells, at 2% and 6.4%, respectively. In contrast, adding IFNβ at later time points, between 8 and 57 hours post-inoculation after the virus had entered the cells and viral gene and protein expression had started, proved less effective, with progressively diminishing impacts (Fig. 6C). These results demonstrate that interferon-beta blocks BKV at an early stage of infection.

### Single-cell transcriptomics reveals a heterogeneous response to BKV infection in endothelial cells

Next, we assessed viral and cellular mRNA abundance at the single-cell level. Two independent single-cell transcriptomic (SCT) studies (Run A and Run B) were performed. VEC from two separate donors (VEC-A and VEC-B, respectively) were mock infected or inoculated with BKV at a multiplicity of infection of 5. Cells were harvested and processed for SCT at 3 days post-infection. After sequencing and quality control steps, the mock- and BKV-infected VEC were computationally combined, and their gene expression profiles were characterized using Seurat to identify clusters visualized using UMAP.

**Fig 7:**
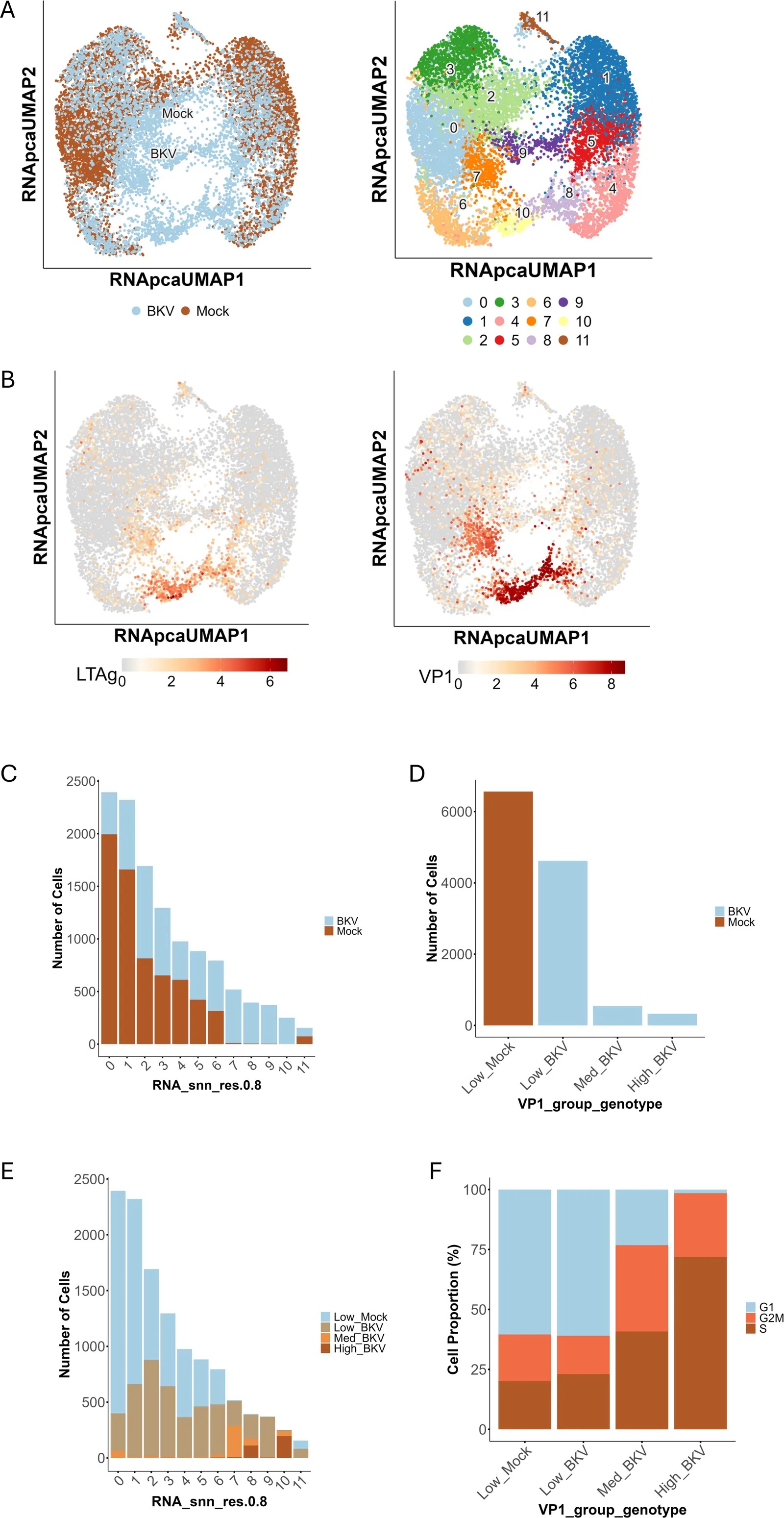
SCT revealed large variations in viral gene expression among BKV-infected VEC from donor A. (**A**) scRNA-seq data of mock- and BKV-inoculated cells (Run A) were analyzed using the Seurat pipeline to identify transcriptionally distinct populations. Each dot represents a single cell. Left panel: UMAP visualization with cells colored by sample condition. Right panel: UMAP visualization showing 12 distinct cell clusters (numbered 0–11) identified based on their gene expression profiles. (**B**) Feature plots of viral gene expression (log normalized) for the early region (LTAg) and the late region (VP1) of the viral genome. (**C**) Compositions of mock- and BKV-infected cells in each cluster. Cluster identities are listed on the x-axis. The number of cells in each cluster is indicated by the y-axis. The composition of mock- (red) vs BKV- (blue) infected cells in each cluster is represented by the corresponding bar. Clusters 7–10 are dominated by BKV-infected cells. (**D**) Number of cells for each VP1 group. (**E**) Same data as in panel C but now colored by VP1 group in each cluster. (**F**) Distribution of cells based on G1, G2/M, and S phases in cell cycle phases across all mock- and BKV-infected cells.

**Fig 8:**
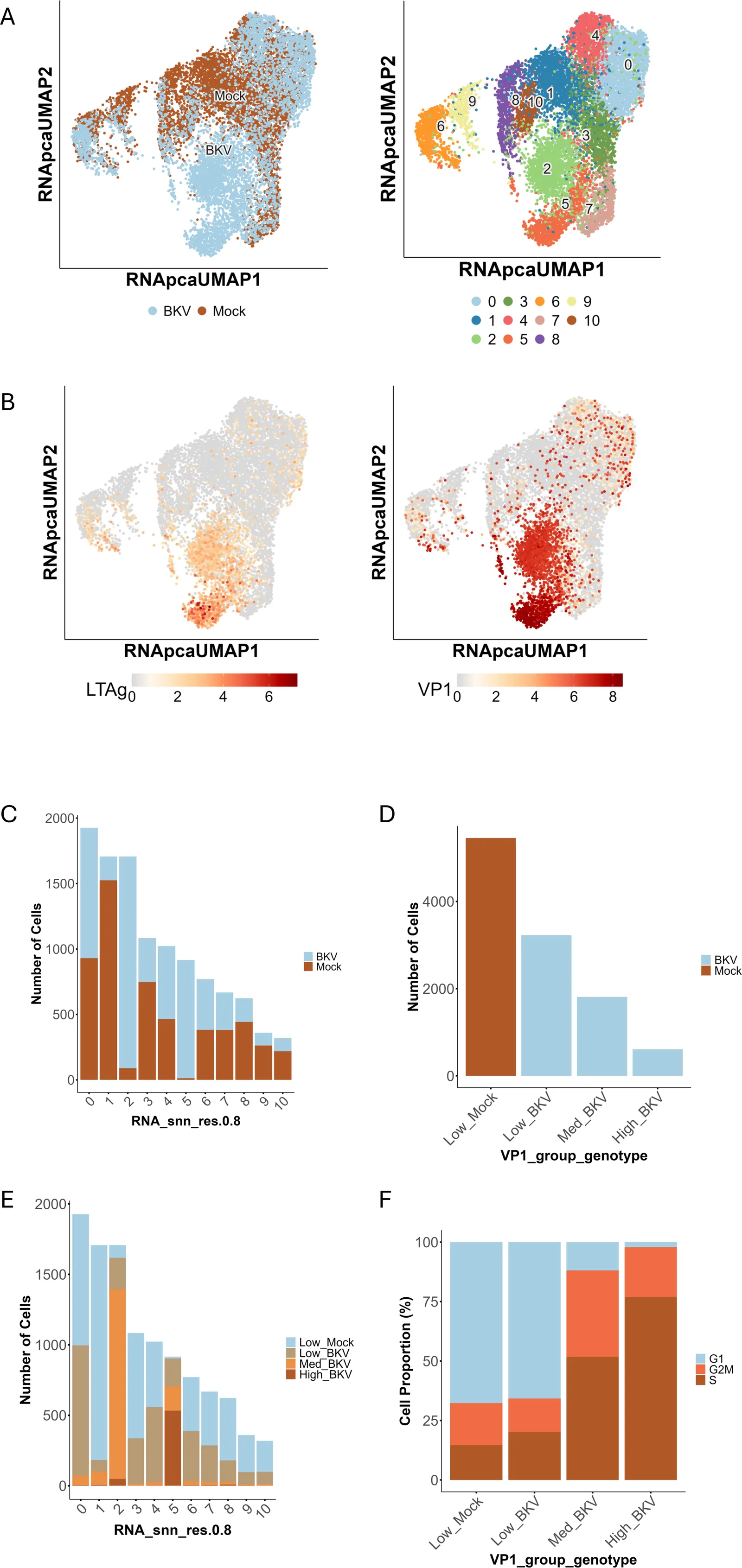
Similar large variations in viral gene expression were reproduced using VEC from donor B. (**A**) scRNA-seq data of mock- and BKV-inoculated cells (Run B) were analyzed using the Seurat pipeline to identify transcriptionally distinct populations. Each dot represents a single cell. Left panel: UMAP visualization with cells colored by sample condition. Right panel: UMAP visualization showing 11 distinct cell clusters (numbered 0–10) identified based on their gene expression profiles. (**B**) Feature plots of viral gene expression (log normalized) for the early region (LTAg) and the late region (VP1) of the viral genome. (**C**) Compositions of mock- and BKV-infected cells in each cluster. Cluster identities are listed on the x-axis. Number of cells in each cluster is indicated by the y-axis. The composition of mock- (red) vs. BKV- (blue) infected cells in each cluster is represented by the corresponding bar. Clusters 2 and 5 are dominated by BKV-infected cells. (**D**) Number of cells for each VP1 group. (**E**) Same data as in panel C but now colored by VP1 group in each cluster. (**F**) Distribution of cells based on G1, G2/M, and S phases in cell cycle phases across all mock- and BKV-infected cells.

In Run A, 12 distinct clusters (0–11) were identified, with a relatively small subset of cells expressing high levels of viral transcripts (Fig. 7A and B). At least one viral read was detected in approximately 55% of BKV-inoculated cells. Seven of the clusters (0–6) were composed of a mixture of mock- and BKV-inoculated cells, while four clusters (7–10) contained almost exclusively BKV-inoculated cells (Fig. 7C). Cluster 11 contained very few cells, and their gene expression profile was indicative of cell death. Run B showed a similar pattern, with 11 clusters, of which 2 were predominantly composed of BKV-inoculated cells (Fig. 8A through C).

Next, the level of viral transcripts across the BKV-inoculated cell population was assessed. For this purpose, we divided cells into one of three groups: low, medium, and high (Fig. 7D and 8D). The high group consists of cells in which 10% or greater of the transcriptome consists of viral reads. The medium group is cells in which at least 1%–10% of the transcripts are viral. The remainder are in the low group. Among BKV-inoculated cells, 84% were in the low group, with the remaining 16% being medium or high. Most of the low group clustered with mock samples, suggesting cells harboring low levels of viral transcripts have little or no impact on the expression of cellular genes (Fig. 7E and 8E). Clusters 7, 8, and 10 of Run A and clusters 2 and 5 of Run B contain most of the cells in the medium and high groups. Finally, we assessed the level of viral transcripts in different stages of the cell cycle (Fig. 7F and 8F). Mock cells as well as BKV-inoculated cells in the low expression group were distributed throughout the cell cycle, with a majority being in G1. In contrast, cells in the medium and high groups had fewer cells in G1, with a majority being in the cycle. Similar associations between levels of viral transcripts and cell cycle stages were observed in Run B, indicating that the observed trends were reproducible across donors.

**Fig 9:**
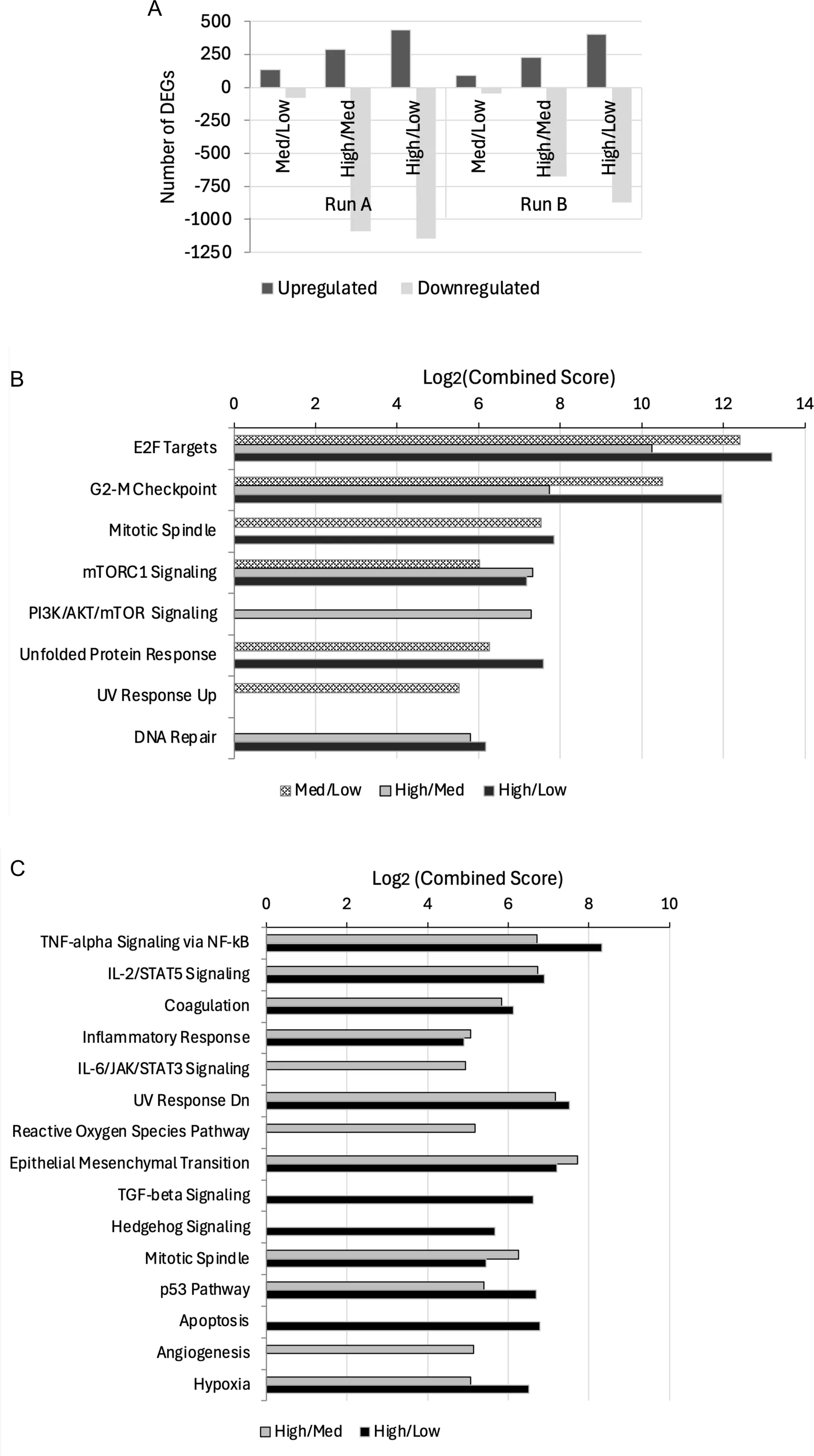
Differentially expressed genes associated with low, medium, and high levels of viral gene expression showed enrichment for specific pathways. (**A**) A summary of the number of DEGs upregulated or downregulated. BKV-infected cells were sorted into three groups based on the abundance of viral reads relative to total reads: Low (cells containing <1% of viral reads), Med (cells containing 1%–10% viral reads), and High (cells containing >10% viral reads). Upregulated and downregulated DEGs (at least twofold difference in mean expression) were identified by comparing average expression of cellular genes across cells in each group for three comparisons: Med/Low, High/Med, and High/Low. (**B**) Pathways associated with upregulated DEGs in the three comparisons. (**C**) Pathways associated with downregulated DEGs in the High/Med and High/Low comparisons. For both panels B and C, the x-axes of the bar graphs represent log2-transformed combined scores of each pathway listed on the y-axes. The combined scores were calculated based on the DEGs associated with the corresponding pathways. Greater combined scores indicate stronger confidence in the predicted pathway. All pathways included in the graph were associated with combined scores of at least 30.

Average expression values for all protein-coding cellular genes were calculated for the low, medium, and high viral expression groups and used to identify differentially expressed genes (DEGs). DEGs were defined as those exhibiting at least a twofold difference in group average expression values (see Materials and Methods for details). In both SCT runs, cells in the high group showed the most upregulated or downregulated DEGs. The medium group showed more upregulated DEGs than downregulated ones, whereas in the high group, downregulated DEGs outnumbered upregulated ones by at least twofold (Fig. 9A). Importantly, dramatic downregulation of cell genes was not evident in cells expressing medium levels of viral transcripts. The common DEGs between Runs A and B were subjected to pathway enrichment analysis, which subsequently revealed distinct cellular processes associated with medium to high levels of viral gene expression. The upregulated DEGs were enriched for processes related to cell proliferation including E2F targets, G2-M checkpoints, and mitotic spindle, with these pathways becoming increasingly enriched as cells progressed from medium to high expressions of viral genes (Fig. 9B). This trend is consistent with prior knowledge of polyomavirus replication, in which successful infection requires host cell cycle activation to support viral DNA replication (20). Additionally, upregulated DEGs showed enrichment in pathways associated with DNA repair, mTOR signaling, UV response, and unfolded protein response. Downregulated DEGs were linked to a broad range of cellular processes, including innate immune response and inflammation response, reactive oxygen species (ROS) signaling, hypoxia, epithelial-to-mesenchymal transition (EMT), and apoptosis (Fig. 9C).

### Single-cell transcriptomic analysis of the innate immune response

**Fig 10:**
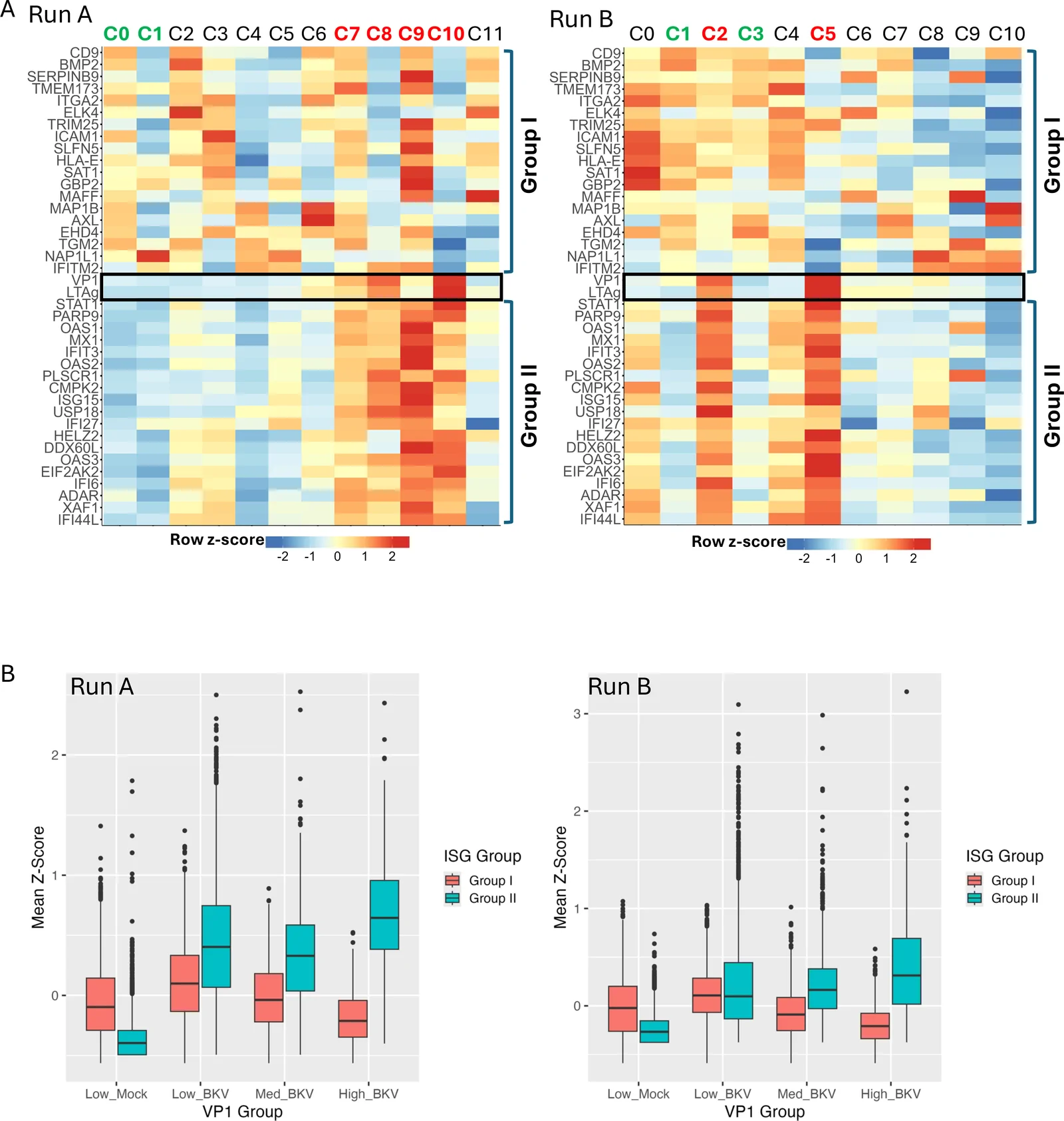
BKV infection leads to distinctive patterns of ISG induction. (**A**) Heatmap representing cluster average expression of ISGs across clusters in Run (left) and Run B (right). Cluster labels in green or red indicate clusters that predominantly contain mock- or BKV-infected cells, respectively. Heatmap colors represent Z-scores across each row (scale bar below the heatmap). Group I and Group II ISGs are indicated with brackets, and VP1 and LTAg rows are highlighted by a horizontal box. (**B**) Boxplots showing mean Z-scores of Group I and Group II ISGs across VP1 expression groups (x-axis) for Run A (left) and Run B (right). Group II ISGs display higher mean Z-scores compared to Group I ISGs as BKV expression increases.

We have shown that the IFN response plays a key role in limiting BKV infection of endothelial cells. Therefore, we focused on characterizing the expression and induction of ISGs in mock- and BKV-infected cells. To accomplish this, cluster average expression values for ISGs and viral genes across clusters were calculated and visualized as a heatmap (Fig. 10A). This analysis revealed two distinct groups of ISGs based on their patterns of expression/induction. The group I ISGs, which included approximately a dozen genes such as *SAT1*, *TGM2*, *CD9*, and *TRIM25*, were prominently expressed or induced in clusters 7 and 9 (Run A) cells with low levels of viral reads. These same ISGs were uninduced/downregulated in clusters 8 and 10 (Run A), cells that showed high BKV gene expression (Fig. 10A, top half of heatmap; Fig. 10B). While some of these ISGs were induced in clusters 0–6 of Run A, most were expressed at a basal level in these clusters. In contrast, Group II ISGs, comprising around 20 genes, such as *OAS1*, *MX1*, *ISG15*, and *IFI6*, were strongly induced in clusters 7–10, clusters predominantly containing BKV-infected cells (Fig. 10A, bottom half of heatmap; Fig. 10B). However, despite robust induction of these ISGs, cluster 8 and 10 cells expressed BKV genes at high levels. Analysis of the mean Z-scores of Group I vs Group II ISGs in both runs further confirmed that downregulation of Group I ISGs was associated with the rise of viral transcripts (Fig. 10B). The expression and induction patterns of Group I ISGs suggest their potential contributions to restriction of BKV infection.

## DISCUSSION

Previous studies have shown that the innate immune response, specifically the IFNβ pathway, is activated by BKV in primary endothelial cells (13, 17). In this manuscript, we show that TIME cells (hTERT-immortalized endothelial cells) respond similarly to BKV as VEC (primary human lung microvascular endothelial cells). IFN transcripts (IFNB1) were detected in BKV-infected cells in bulk RNA-seq, and IFNβ protein was detected in infected cell supernatants, but only at the late stages of infection (Fig. 2). This indicates that neither incoming virus particles nor low levels of LT induce an IFN response. Rather, IFN pathway activation occurs either simultaneously with or soon after the onset of viral DNA replication, late gene expression, and/or the assembly of progeny virions.

BKV establishes long-term persistent infection in both TIME and VEC. This is characterized by persistent viral protein expression (Fig. 4), viral genome replication (Fig. 5A), and release of infectious progeny (Fig. 5B). Evidence presented in this study suggests that IFNβ limits viral infection in TIME, likely contributing to the persistent infection. We show that the addition of purified IFNβ to cultures prior to infection efficiently blocks BKV infection. Similar inhibition is also observed when IFN is added at 3 hours post-inoculation. Significant inhibition is also seen when IFNβ is added 8 hours post-infection (Fig. 6). The addition of IFN at later times post-infection, after the onset of viral DNA replication and late gene expression, has little or no effect. These results suggest that IFN blocks BKV at an early stage of infection, most likely uncoating, nuclear entry, or early viral gene transcription.

Among cells infected at an MOI of 5, only 30%–40% showed positive staining of LT using immunofluorescence (Fig. 1) and about 16% expressed medium to high levels of viral transcripts based on SCT (Fig. 7 and 8). We conclude that only a subset of BKV-inoculated cells are undergoing a full productive infection. SCT result is an underestimate of the amount of viral transcripts. So the absence of viral transcripts either means none are present, or they are present at a level below the detection limits. These trends are consistent with what is observed with BKV-infected RPTE cells, although the levels are somewhat lower (21). Furthermore, as in RPTE, nearly all infected endothelial cells containing the highest levels of viral transcripts (medium and high expression groups) are in S or G2M (Fig. 7 and 8) (21). Pathway analysis of upregulated DEGs further supports robust activation of the cell cycle in the medium and high viral gene expression cell groups (Fig. 9B). In contrast, most cells exhibiting low levels of viral gene expression are growth arrested. These results favor a model in which high levels of viral gene expression occur only in cells that have entered the cell cycle. Again, these same observations occur in RPTE and are consistent with studies showing that high LT levels require cells to be in S phase. In addition, SCT data show that BKV-inoculated cells expressing low levels of viral transcripts had similar cellular gene expression profiles to mock cells (Fig. 7, Run A: clusters 0–6). In general, these cells expressed basal levels of genes regulated by E2F transcription factors, consistent with most cells being growth arrested.

Increases in both IFNB1 transcripts and IFNβ protein following BKV infection were detected by bulk RNA-seq and ELISA, respectively (Fig. 2). In SCT studies, IFN transcripts (UMI count ≤2) were detected in only approximately 0.1% of BKV-infected cells (7 out of 6,043), suggesting that only a rare subpopulation produces IFNB1 transcripts at detectable levels. BKV-infected cells containing low levels of viral transcripts do not show consistent patterns of ISG induction. Thus, the interferon response alone does not appear to be responsible for the low level of viral infection. Rather, as has been observed for BKV infections of RPTE, the repression of viral productive infection appears to be due to the lack of cell cycle activation. With that said, the heterogeneous levels of ISG induction across infected cells coupled with the levels of viral gene transcripts suggest that a subset of these ISGs may act to limit BKV infection (Fig. 10). Particularly, the Group I ISGs such as *SAT1*, *TGM2*, *CD9*, and *TRIM25* are upregulated in clusters of mainly BKV-infected cells containing low levels of viral transcripts. Identification of the key restrictive ISGs awaits further investigation. In addition, pathway analysis suggests that downregulated DEGs in the medium and high viral expression groups of cells are mostly involved in inflammatory response, cell death, and epithelial-to-mesenchymal transition (Fig. 9C). Effects of these DEGs and their associated pathways on limiting BKV infection remain to be verified and further characterized.

## MATERIALS AND METHODS

### Cells and viral stock

VEC (primary human lung microvascular endothelial cells, catalog number CC2527) from three separate donors (A, B, and C) were purchased from Lonza, and TIME (hTERT-immortalized human neonatal foreskin microvascular endothelial cells [22], catalog number CRL-4025) were purchased from ATCC. Both cell types were cultured based on the manufacturer’s recommendations. VEC from donors A and B were used for the single-cell transcriptomics studies, whereas VEC from donor C was used in all other experiments presented in this manuscript.

The BKV strain used in all experiments was Dunlop, prepared as described previously (13, 23). Infections were conducted at an MOI of 5. For inoculation, cells were pre-cooled for 15 minutes at 4°C before being exposed to BKV stock diluted in fresh growth medium. During the 1-hour incubation, plates were gently rocked every 15 minutes. After incubation, the inoculum was removed and replaced with the original growth medium. Mock-infected cells underwent the same procedure, except no BKV stock was added.

BKV viral stock was grown in 293TT cells at an MOI of 0.1 in a 10 cm dish, and once confluent, cells were passaged into T150 flasks. 293TT cells were maintained in complete growth medium containing DMEM + 10% FBS (heat inactivated) with gentamycin (50 µg/mL) and amphotericin (0.25 µg/mL), and they were cultured in DMEM + 2% FBS (heat inactivated) with 10 mM MgSO4, gentamycin, and amphotericin after passage into flasks. Once cells began to detach from the flasks around 2 weeks, cells and supernatant were collected. Cell debris and supernatant were separated by centrifugation at 8,000 × *g* for 30 minutes in an SLA-3000 rotor (Sorvall). The supernatant (S1) was collected and frozen until viral prep. The pellet was resuspended in 10 mL Buffer A (10 mM HEPES, pH 8, 1 mM CaCl2, 1 mM MgCl2, and 5 mM KCl). A portion (100 µL) was used for miniprep to confirm Dunlop stock, and the remainder was frozen (S2). S1 and S2 were thawed, and S2 was resuspended with a 21G needle followed by pH adjustment to 6 with HEPES pH 5.5. Ten units of neuraminidase (Sigma) was added to S2 and incubated at room temperature for 1.5 hours, inverting every 20 minutes. S1 was centrifuged at 8,000 × *g* for 30 minutes in an SLA-3000 rotor, and the supernatant was collected and stored on ice. After the NA treatment, the pH of S2 was adjusted to 7.4 using HEPES pH 8, followed by incubation at 42°C for 5 minutes. S2 was then centrifuged at 16,000 × *g* for 5 minutes in an SS-34 rotor (Sorvall). The pellet (P2) was resuspended in 5 mL Buffer A + 0.1% DOC and vortexed. P2 was incubated at room temperature for 15 minutes, vortexing every 5 minutes. P2 was centrifuged at 16,000 × *g* for 5 minutes, and the final supernatant (S3) was combined with S1 and S2 in 38.5 mL open-top thin-wall ultra-clear tubes (Beckman) on a 4.5 mL 20% sucrose in Buffer A cushion. Virus was pelleted by centrifugation at 25,000 rpm for 3 hours in an SW-28 rotor (Beckman). The pellet was resuspended in 700 µL Buffer A and frozen at −20°C in 70 µL aliquots.

Ten microliters of viral stock was treated with 1 µL Turbo DNase and 20 µL 1× Turbo DNase buffer diluted to 200 µL in Buffer A for 20 minutes at 37°C. BKV genomic DNA was extracted using the DNeasy blood and tissue kit (Qiagen), and Dunlop viral stock was confirmed by gel electrophoresis. Viral stocks were quantified as described (2) by absolute quantification PCR (qPCR) using plasmid BKV, containing the BKV genome, as the standard. The following primers were used to bind the late region of the BKV genome: BKV VP1 (5′CAGTTACAGCCTCCCACATCAGTAG and 5′GCCCAGAGAGAAAAATGCTTCC). Concentration was determined by qPCR with Power SYBR Green RNA-to-CT 1-step kit (Thermo Fisher) in triplicate. Genome copies were calculated to determine how much viral stock to use for MOI 5 infections.

Viral titer was calculated by inoculating hTERT-RPTE4 cells with supernatant. hTERT-RPTE4 cells were fixed at 2 dpi and stained for LT (Pab416 antibody, mouse monoclonal, 1:20). % LT-positive cell was quantified and compared to nuclear, or DAPI, staining. Viral titer was calculated with the following: Titer (FFU/mL) = (number of cells * % LT-positive cells)/(volume of inoculum).

### IFNβ

Recombinant human IFNβ (R&D Systems) was reconstituted in ddH20 to a specific activity of 5.6 × 10^4^ IU/µL and a concentration of 0.2 µg/µL. 10 U/mL, 30 U/mL, or 100 U/mL IFNβ was added to culture supernatant at the indicated time points.

### Immunofluorescence and microscopy

Immunofluorescence was performed with the following primary antibodies: Pab416 (LT antibody, mouse monoclonal, 1:20) (24), anti-VP1 (Abnova, mouse monoclonal, 1:400), rabbit anti-STAT Y701 (Cell Signaling, 1:400), and rabbit anti-IRF3 (Cell Signaling, 1:400). The following secondary antibodies were used: goat anti-mouse Alexa Fluor 488 or 568 (Thermo Fisher Scientific, 1:400) and goat anti-rabbit Alexa Fluor 488 (Thermo Fisher Scientific, 1:400). Primary antibodies were incubated overnight at 4°C, while secondary antibodies were incubated at room temperature for 1 hour.

Coverslips were treated with 0.01% poly D lysine before plating. Cells were fixed on coverslips in 4% formaldehyde for 20 minutes at room temperature, followed by three 5-minute washes with 1× PBS. Cells were blocked with 5% NGS, 0.3% Triton X-100, and 1× PBS for 1 hour at room temperature. Primary and secondary antibodies were incubated in 1% BSA, 0.3% Triton X-100, and 1× PBS overnight at 4°C or 1 hour at room temperature, respectively. Coverslips were washed three times for 5 minutes with 1× PBS after primary and secondary antibody incubations. After the final wash, coverslips were dried and mounted on slides with Pro-Long Gold Antifade reagent with DAPI (Thermo Fisher Scientific). Coverslips were imaged with a Zeiss AX10 microscope with fluorescence filters.

Before blocking, rabbit anti-STAT Y701 coverslips were methanol permeabilized with 100% ice-cold MeOH for 10 minutes at 4°C, followed by three 5 minute-washes with 1× PBS on ice. These coverslips were imaged with Leica SP8 Confocal. To quantify % positive cells, the ImageJ cell counter point tool was used.

### RNA isolation and RT-qPCR

TRIzol (Thermo Fisher) was used to extract RNA from cells in 6-well plates. RNA isolation was completed with Direct-Zol RNA Miniprep (Zymo Research) according to the manufacturer’s instructions.

RT-qPCR was performed with Power SYBR Green RNA-to-CT 1-Step qRT-PCR Kit (Thermo Fisher Scientific). Samples were analyzed in triplicate with 200 ng RNA and 200 nM primers according to the kit instructions. Values were normalized to the endogenous control GAPDH and a BKV-infected 0 hpi control. The following primer sets were used: BKV LT (5′GAGTAGCTCAGAGGTGCCAACC and 5′CATCACTGGCAAACATATCTTCATGGC), BKV VP1 (5′CAGTTACAGCCTCCCACATCAGTAG and 5′GCCCAGAGAGAAAAATGCTTCC), and GAPDH (5′AAGGTGAAGGTCGGAGTCAA and 5′AATGAAGGGGTCATTGATGG).

### Bulk RNA-seq

Previously published VEC RNA-seq fastq files were obtained (13) and reanalyzed here using the same pipeline as described below. For TIME RNA-seq, RNA was extracted as described above. Quality control (Qubit and TapeStation) and library preparation were done by the Health Sciences Sequencing Core (HSSC) at UPMC Children’s Hospital of Pittsburgh (https://www.advancedgenomics.pitt.edu/health-sciences-sequencing-core-upmc-childrens-hospital-pittsburgh) using the Illumina Stranded mRNA Prep kit. Libraries were sequenced on a NextSeq 2000 to an approximate depth of 60 million paired-end reads (58 bp reads).

Raw fastq files for both VEC and TIME were processed with the nfcore/rnaseq pipeline (version 3.3; https://nf-co.re/rnaseq/3.3). Reads were aligned to a hybrid reference of the human genome (Gencode GRCh38.primary_assembly.genome.fa) and the BKV genome (NC_001538.1) with gene annotations from Gencode V38 GTF. Coverage maps for the BKV genome (NC_001538.1) were generated using the Integrative Genomics Viewer (version 2.16.0).

### DNA isolation, restriction digestion, and gel electrophoresis

BKV genomic DNA was isolated with the QIAprep Spin Miniprep kit (QIAGEN). Cells were scraped and collected from 6-well plates in 220 µL 1× PBS. Fifty microliters of buffer P1 was added before following the manufacturer’s protocol. DNA was eluted in ddH20.

BKV genomic DNA (GenBank accession number KP412983) was linearized by restriction digestion with BamHI-HF (Fisher Scientific). Twenty microliters of reactions was made with 10× FastDigest Green Buffer, BamHI-HF, DNA, and nuclease-free water according to the manufacturer’s instructions. pBKV was digested with BAMHI as a positive control. Reactions were incubated at 37°C for 40 minutes, followed by an 80°C heat inactivation for 5 minutes. The digested DNA was run on a 1% agarose gel containing 1× SYBR Gold Nucleic Acid Gel Stain (Thermo Fisher Scientific) for visualization of DNA. The amount of DNA in both the positive control (BamHI-digested pBKV) and negative control (undigested pBKV) was 50 ng. Digested miniprep DNA isolated from 50,000 mock-infected or BKV-infected cells (cell numbers estimated based on initial seeding density) was loaded.

### ELISA

IFNβ production was measured with the Human Luminex Discovery Kit (R&D Systems) in duplicate from mock- and BKV-infected culture supernatants. Samples were stored at −80°C until processing at the UPMC Cancer Proteomics Facility: Luminex Core Laboratory.

### Heatmaps

In Fig. 2, for TIME and VEC, TPM values from BKV-infected samples at 72 hpi were filtered to keep genes with TPM ≥ 10, and the top 50 ISGs by TPM were selected. The two top 50 lists were combined into a union set (32 ISGs intersection and 18 unique to each). Log2 TPM values for mock- and BKV-infected TIME and VEC at 72 hpi were then plotted as a heatmap using pheatmap in R without clustering.

In Fig. 10, heatmaps of selected Group I and Group II ISGs and BKV gene expression across single-cell clusters were generated using the ShinyCell R package. Average expression per cluster was z-score scaled across clusters for each gene.

### SCT sample processing and sequencing

Two separate SCT experiments termed Run A and Run B were conducted using VEC from donors A and B (Run A: VEC-A and Run B: VEC-B). In both experiments, mock- and BKV- (MOI of 5) infected cells were harvested at 3 dpi. We processed and analyzed between 6,000 and 7,000 cells per sample for both runs. The method for library construction and sequencing was performed as described in An et al. (21).

### scRNA-seq data processing and analysis

Fastq files were processed with Indrops software (https://github.com/indrops/indrops) as previously described in An et al. (21). Raw UMI count tables contained 41,858 genes, and the number of cells per sample is as follows: Run A Mock 6,954 and BKV 6,043, and Run B Mock 6,005 and BKV 6,247. Cell filtering cutoffs were determined using the “scater” R package (25) on each sample individually. Cutoffs for minimum and maximum UMI counts, minimum features (genes), and maximum mitochondrial percentage were determined using a median absolute deviation cutoff of 2. Then two independent Seurat analyses were performed. A Seurat object was created from the two samples in Run A (Mock and BKV) and another object was created from the two samples in Run B (Mock and BKV). Indrops raw UMI count data were loaded with Seurat (v4.3) (26), and cells were filtered based on the scater cutoffs. This process removed 6%–10% of cells across all four samples. Genes expressed in fewer than 50 cells were excluded, resulting in 15.6K and 14.4K genes remaining in Run A and Run B, respectively.

Using Seurat on the filtered cells, we performed data normalization and log transformation with the NormalizeData function. Subsequently, variable features were identified by using FindVariableFeatures (selection.method = “mean.var.plot”). These variable genes underwent scaling with ScaleData with regression on total UMI count (nCount_RNA). Principal component analysis (PCA) was then conducted on the variable genes using RunPCA. To determine the optimal number of PCA dimensions for downstream analyses, the PC that accounted for a minimum of 30% of the cumulative PCA standard deviation was selected. Cell cycle phases were assigned using Seurat’s CellCycleScoring() function. UMAP was used for visualization (RunUMAP), and clustering was performed using Seurat’s FindClusters function with a resolution of 0.8, which resulted in 12 and 11 clusters for Run A and Run B, respectively. To characterize cluster-specific gene expression patterns, the average expression of each gene for each cluster was determined with the AverageExpression function by setting group.by = “RNA_snn_res.0.8.” VP1 transcript group analysis was performed as previously described (23). Average gene expression for each of the three groups was calculated using the Seurat function AverageExpression.

Three sets of DEGs were identified by comparing average gene expression levels across the low (Low: cells with <1% viral transcripts), medium (Med: cells with 1%–10% viral transcripts), and high (High: cells with >10% viral transcripts) BKV expression groups. The DEGs were as follows: (i) upregulated genes in the Med group relative to the Low group (46 genes commonly upregulated in both runs), (ii) upregulated genes in the High group relative to the Med group (94 genes commonly upregulated in both runs), and (iii) downregulated genes in the High group relative to the Med group (378 genes commonly downregulated in both runs). All DEGs met a minimum threshold of a twofold change for significant differential expression. The median average gene expression values across all six groups (Run A: Low, Med, and High; Run B: Low, Med, and High) ranged from 0.13 to 0.17. To ensure reliable fold-change calculations, gene expression values for the numerators were set to a minimum of 0.3 for upregulated genes in Med vs Low and High vs Med, while for downregulated genes in High vs Med, the denominators were set to at least 0.3. The common DEGs between the two runs were analyzed using the Enrichr platform (https://maayanlab.cloud/Enrichr/) for pathway enrichment (27–29) based on the hallmark gene sets from the Molecular Signature Database (MSigDB hallmark 2020) (30). Representative pathways with adjusted *P*-values < 0.05 and combined scores greater than 30 or less than −30 were summarized in a bar graph.

### Software versions

R version 4.2.3 (2023-03-15) was used with the following packages: ggplot 3.5.1 (Box plots), scater 1.26.1 (SCT quality control), Seurat 4.3.0 (SCT analysis pipeline), and ShinyCell 2.1.0 (UMAP plots, barplots, and ISG expression heatmap in Fig. 10).

## Data Availability

The RNA-seq data generated and analyzed in this study have been deposited in the National Center for Biotechnology Information (NCBI) Gene Expression Omnibus (GEO) database and are accessible through GEO Series accession number GSE336437 (nih.gov).
